# Diaqua­[(*E*)-2-(2-oxidobenzyl­idene­amino)-2-phenyl­acetato]zinc(II) dimethyl sulfoxide monosolvate

**DOI:** 10.1107/S1600536809020741

**Published:** 2009-06-06

**Authors:** Jun You, Bo Liu, Yu-Jing Pang, Qing-Cui Wu

**Affiliations:** a School of Chemistry and Environment Engineering, Harbin University of Science and Technology, Harbin 150040, People’s Republic of China

## Abstract

In the title compound, [Zn(C_15_H_11_NO_3_)(H_2_O)_2_]·C_2_H_6_OS, the Zn(II) ion is coordinated by two O atoms and one N atom of the deprotonated chelate ligand and two water mol­ecules in a distorted trigonal bipyramidal coordination environment. A linear supra­molecular structure built from O—H⋯O hydrogen bonds runs parallel to [100].

## Related literature

For the synthesis of (*E*)-2-(2-hydroxy­benzyl­ideneamino)-2-phenyl­acetic acid, see Audriceth *et al.* (1954 ▶). For a related zinc complex, see: You *et al.* (2008 ▶).
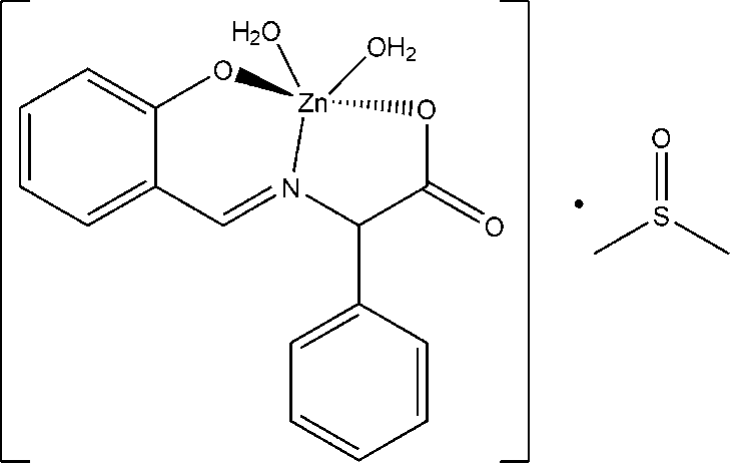


## Experimental

### 

#### Crystal data

[Zn(C_15_H_11_NO_3_)(H_2_O)_2_]·C_2_H_6_OS
*M* *_r_* = 432.78
Triclinic, 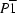

*a* = 7.331 (4) Å
*b* = 9.318 (5) Å
*c* = 14.578 (9) Å
α = 81.91 (2)°
β = 81.37 (2)°
γ = 80.18 (2)°
*V* = 963.4 (9) Å^3^
*Z* = 2
Mo *K*α radiation
μ = 1.42 mm^−1^
*T* = 291 K
0.19 × 0.15 × 0.13 mm

#### Data collection

Rigaku R-AXIS RAPID diffractometer
Absorption correction: multi-scan (*ABSCOR*; Higashi, 1995 ▶) *T*_min_ = 0.777, *T*_max_ = 0.837
9415 measured reflections
4331 independent reflections
3751 reflections with *I* > 2σ(*I*)
*R*_int_ = 0.024

#### Refinement

*R*[*F* ^2^ > 2σ(*F* ^2^)] = 0.033
*wR*(*F* ^2^) = 0.098
*S* = 1.15
4331 reflections
237 parameters
H-atom parameters constrained
Δρ_max_ = 0.48 e Å^−3^
Δρ_min_ = −0.28 e Å^−3^

### 

Data collection: *RAPID-AUTO* (Rigaku, 1998 ▶); cell refinement: *RAPID-AUTO*; data reduction: *CrystalStructure* (Rigaku/MSC, 2002 ▶); program(s) used to solve structure: *SHELXS97* (Sheldrick, 2008 ▶); program(s) used to refine structure: *SHELXL97* (Sheldrick, 2008 ▶); molecular graphics: *SHELXTL* (Sheldrick, 2008 ▶); software used to prepare material for publication: *SHELXL97*.

## Supplementary Material

Crystal structure: contains datablocks global, I. DOI: 10.1107/S1600536809020741/ng2591sup1.cif

Structure factors: contains datablocks I. DOI: 10.1107/S1600536809020741/ng2591Isup2.hkl

Additional supplementary materials:  crystallographic information; 3D view; checkCIF report

## Figures and Tables

**Table 1:** Selected geometric parameters (Å, °)

N1—Zn1	2.0305 (19)
O1—Zn1	2.1047 (17)
O3—Zn1	1.9742 (18)
O4—Zn1	2.009 (2)
O5—Zn1	2.0015 (19)

**Table 2:** Hydrogen-bond geometry (Å, °)

*D*—H⋯*A*	*D*—H	H⋯*A*	*D*⋯*A*	*D*—H⋯*A*
O4—H23⋯O6	0.85	1.90	2.725 (3)	164
O4—H24⋯O2^i^	0.85	1.88	2.695 (3)	160
O5—H21⋯O2^ii^	0.85	1.81	2.629 (2)	161
O5—H22⋯O6^iii^	0.85	1.93	2.742 (3)	159

Symmetry codes: (i) 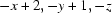; (ii) 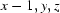; (iii) 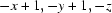.

**Fig. 1.**
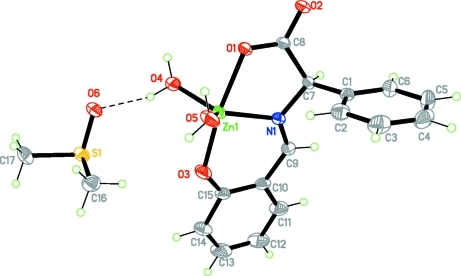
The molecular structure of (I), showing displacement ellipsoids at the 30% probability level for non-H atoms.

**Fig. 2.**
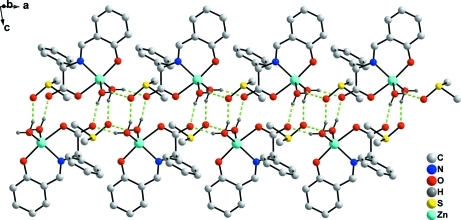
A partial packing view, showing the one-dimensional tubal supramolecular structure. H atoms not involved in hydrogen bonds have been omitted for clarity.

## supplementary crystallographic information

### Comment

The continuous interest in designing and making novel Schiff base ligand and transition-metal complexes have persisted because of their impressive catalytic property. Recently, our group have reported a Schiff base and Zn(II) complex (You *et al.*, 2008). As the continually working, we report a new title Schiff base complex, herein, synthesized by the reaction of (*E*)-2-(2-hydroxybenzylideneamino)-2-phenylacetic acid and Zn(OAc)_2_.

As shown in Fig. 1, Zn^II^ ion is five-coordinate in a slightly distorted trigonal-bipyramidal coordination environment, two water molecules and one N formed the equatorial plane and two deprotonated O atoms take up the apices positions.

The cocrystalized dimethylsulfoxide molecules link the discrete coordinate compound to a one-dimensional tubal supramolecular structure, *via* the O—H···O hydrogen bonds parallel to [100] (Fig. 2).

### Experimental

(*E*)-2-(2-hydroxybenzylideneamino)-2-phenylacetic acid was prepared of 2-amino-2-phenylacetic acid and 2-hydroxybenzaldehyde in aqueous solution (Audriceth *et al.*, 1954). (*E*)-2-(2-hydroxybenzylideneamino)-2-phenylacetic acid (0.255 g, 1 mmol) and Zn(OAc)_2_ (0.190 g, 1 mmol) dissolved in hot aqueous solution (20 ml) then refluxed for 1 huor. After cooling to room temperature the solution was filtered, the residue was recrystaled in DMSO and methanol (10/1, V/V) solution, several days latter, a suitable for X-ray diffraction yellow crystal was obtained.

### Refinement

H atoms bound to C atoms were placed in calculated positions and treated as riding on their parent atoms, with C—H = 0.93 Å (aromatic C), C—H = 0.98 Å (methylene C), C—H = 0.96 Å (methyl C) and with *U*_iso_(H) = 1.2Ueq(C). Water H atoms were initially located in a difference Fourier map, but they were treated as riding on their parent atoms with O—H = 0.85 Å and with with *U*_iso_(H) = 1.5*U*_eq_(O).

### Crystal data

**Table:**

[Zn(C_15_H_11_NO_3_)(H_2_O)_2_]·C_2_H_6_OS	*Z* = 2
*M**_r_* = 432.78	*F*(000) = 448
Triclinic, *P*1	*D*_x_ = 1.492 Mg m^−^^3^
Hall symbol: -P 1	Mo *K*α radiation, λ = 0.71073 Å
*a* = 7.331 (4) Å	Cell parameters from 8052 reflections
*b* = 9.318 (5) Å	θ = 3.0–27.5°
*c* = 14.578 (9) Å	µ = 1.42 mm^−^^1^
α = 81.91 (2)°	*T* = 291 K
β = 81.37 (2)°	Block, colorless
γ = 80.18 (2)°	0.19 × 0.15 × 0.13 mm
*V* = 963.4 (9) Å^3^	

### Data collection

**Table:**

Rigaku R-AXIS RAPID diffractometer	4331 independent reflections
Radiation source: fine-focus sealed tube	3751 reflections with *I* > 2σ(*I*)
graphite	*R*_int_ = 0.024
ω scan	θ_max_ = 27.5°, θ_min_ = 3.0°
Absorption correction: multi-scan (*ABSCOR*; Higashi, 1995)	*h* = −9→8
*T*_min_ = 0.777, *T*_max_ = 0.837	*k* = −12→12
9415 measured reflections	*l* = −18→18

### Refinement

**Table:**

Refinement on *F*^2^	Primary atom site location: structure-invariant direct methods
Least-squares matrix: full	Secondary atom site location: difference Fourier map
*R*[*F*^2^ > 2σ(*F*^2^)] = 0.033	Hydrogen site location: inferred from neighbouring sites
*wR*(*F*^2^) = 0.098	H-atom parameters constrained
*S* = 1.15	*w* = 1/[σ^2^(*F*_o_^2^) + (0.0512*P*)^2^ + 0.2263*P*] where *P* = (*F*_o_^2^ + 2*F*_c_^2^)/3
4331 reflections	(Δ/σ)_max_ = 0.001
237 parameters	Δρ_max_ = 0.48 e Å^−^^3^
0 restraints	Δρ_min_ = −0.28 e Å^−^^3^

### Special details

**Table:**

Geometry. All e.s.d.'s (except the e.s.d. in the dihedral angle between two l.s. planes) are estimated using the full covariance matrix. The cell e.s.d.'s are taken into account individually in the estimation of e.s.d.'s in distances, angles and torsion angles; correlations between e.s.d.'s in cell parameters are only used when they are defined by crystal symmetry. An approximate (isotropic) treatment of cell e.s.d.'s is used for estimating e.s.d.'s involving l.s. planes.
Refinement. Refinement of *F*^2^ against ALL reflections. The weighted *R*-factor *wR* and goodness of fit *S* are based on *F*^2^, conventional *R*-factors *R* are based on *F*, with *F* set to zero for negative *F*^2^. The threshold expression of *F*^2^ > σ(*F*^2^) is used only for calculating *R*-factors(gt) *etc*. and is not relevant to the choice of reflections for refinement. *R*-factors based on *F*^2^ are statistically about twice as large as those based on *F*, and *R*- factors based on ALL data will be even larger.

### Fractional atomic coordinates and isotropic or equivalent isotropic displacement parameters (Å^2^)

**Table:**

	*x*	*y*	*z*	*U*_iso_*/*U*_eq_	
C1	0.9379 (3)	0.7675 (2)	0.26445 (14)	0.0316 (4)	
C2	0.7789 (4)	0.8689 (3)	0.24967 (18)	0.0451 (5)	
H2	0.6891	0.8462	0.2176	0.054*	
C3	0.7553 (5)	1.0041 (3)	0.2831 (2)	0.0589 (7)	
H3	0.6491	1.0719	0.2736	0.071*	
C4	0.8891 (5)	1.0382 (3)	0.3305 (2)	0.0671 (9)	
H4	0.8718	1.1284	0.3535	0.080*	
C5	1.0461 (5)	0.9402 (3)	0.3436 (2)	0.0612 (8)	
H5	1.1362	0.9643	0.3749	0.073*	
C6	1.0726 (4)	0.8041 (3)	0.31051 (17)	0.0457 (6)	
H6	1.1806	0.7379	0.3193	0.055*	
C7	0.9648 (3)	0.6175 (2)	0.23069 (14)	0.0286 (4)	
H7	1.0767	0.5582	0.2531	0.034*	
C8	0.9913 (3)	0.6342 (2)	0.12286 (14)	0.0287 (4)	
C9	0.7835 (3)	0.4801 (2)	0.34886 (15)	0.0339 (4)	
H9	0.8721	0.4896	0.3862	0.041*	
C10	0.6363 (3)	0.3983 (2)	0.39195 (15)	0.0356 (5)	
C11	0.6505 (4)	0.3309 (3)	0.48364 (17)	0.0521 (6)	
H11	0.7511	0.3425	0.5124	0.063*	
C12	0.5219 (5)	0.2490 (4)	0.5320 (2)	0.0635 (8)	
H12	0.5353	0.2044	0.5923	0.076*	
C13	0.3711 (5)	0.2339 (4)	0.4894 (2)	0.0655 (8)	
H13	0.2828	0.1781	0.5217	0.079*	
C14	0.3488 (4)	0.2995 (3)	0.4004 (2)	0.0559 (7)	
H14	0.2444	0.2886	0.3743	0.067*	
C15	0.4808 (3)	0.3834 (3)	0.34729 (15)	0.0392 (5)	
C16	0.1636 (4)	0.2801 (3)	0.1543 (2)	0.0527 (6)	
H16A	0.2255	0.3352	0.1877	0.079*	
H16B	0.0506	0.2573	0.1919	0.079*	
H16C	0.1341	0.3372	0.0969	0.079*	
C17	0.1686 (4)	0.0495 (3)	0.0617 (2)	0.0495 (6)	
H17A	0.1290	0.1261	0.0143	0.074*	
H17B	0.0612	0.0201	0.1014	0.074*	
H17C	0.2381	−0.0330	0.0327	0.074*	
N1	0.8040 (2)	0.54080 (19)	0.26458 (12)	0.0301 (4)	
O1	0.8636 (2)	0.60996 (18)	0.08213 (10)	0.0366 (3)	
O2	1.1404 (2)	0.67381 (19)	0.08264 (11)	0.0400 (4)	
O3	0.4514 (2)	0.4435 (2)	0.26298 (12)	0.0505 (4)	
O4	0.6986 (3)	0.3510 (2)	0.09429 (13)	0.0581 (5)	
H23	0.6190	0.2942	0.0951	0.087*	
H24	0.7571	0.3634	0.0395	0.087*	
O5	0.4726 (2)	0.7027 (2)	0.11810 (12)	0.0471 (4)	
H21	0.3578	0.6969	0.1193	0.071*	
H22	0.5174	0.7309	0.0626	0.071*	
O6	0.4740 (2)	0.16387 (19)	0.06180 (12)	0.0447 (4)	
S1	0.31254 (8)	0.11488 (6)	0.12970 (4)	0.03825 (14)	
Zn1	0.63958 (3)	0.52441 (3)	0.167315 (16)	0.03534 (10)	

### Atomic displacement parameters (Å^2^)

**Table:**

	*U*^11^	*U*^22^	*U*^33^	*U*^12^	*U*^13^	*U*^23^
C1	0.0338 (10)	0.0317 (10)	0.0280 (9)	−0.0083 (8)	0.0023 (8)	−0.0019 (8)
C2	0.0419 (13)	0.0445 (13)	0.0463 (13)	−0.0040 (11)	−0.0015 (10)	−0.0040 (11)
C3	0.0648 (18)	0.0380 (13)	0.0642 (18)	0.0045 (13)	0.0099 (14)	−0.0074 (13)
C4	0.104 (3)	0.0411 (15)	0.0562 (17)	−0.0236 (17)	0.0161 (17)	−0.0183 (13)
C5	0.080 (2)	0.0554 (17)	0.0589 (17)	−0.0311 (16)	−0.0067 (15)	−0.0191 (14)
C6	0.0504 (14)	0.0492 (14)	0.0420 (13)	−0.0155 (12)	−0.0069 (11)	−0.0102 (11)
C7	0.0240 (9)	0.0327 (10)	0.0301 (10)	−0.0082 (8)	−0.0031 (7)	−0.0031 (8)
C8	0.0257 (9)	0.0303 (10)	0.0296 (9)	−0.0069 (8)	0.0021 (7)	−0.0040 (8)
C9	0.0360 (11)	0.0367 (11)	0.0287 (10)	−0.0072 (9)	−0.0026 (8)	−0.0023 (8)
C10	0.0412 (12)	0.0338 (11)	0.0285 (10)	−0.0080 (9)	0.0059 (9)	−0.0009 (8)
C11	0.0544 (15)	0.0632 (16)	0.0338 (12)	−0.0107 (13)	0.0012 (11)	0.0063 (11)
C12	0.070 (2)	0.071 (2)	0.0398 (14)	−0.0145 (16)	0.0081 (13)	0.0177 (13)
C13	0.073 (2)	0.0637 (18)	0.0534 (16)	−0.0298 (16)	0.0221 (15)	0.0079 (14)
C14	0.0502 (15)	0.0702 (18)	0.0470 (14)	−0.0287 (14)	0.0107 (12)	−0.0001 (13)
C15	0.0409 (12)	0.0429 (12)	0.0321 (11)	−0.0141 (10)	0.0088 (9)	−0.0035 (9)
C16	0.0515 (15)	0.0485 (14)	0.0578 (16)	−0.0065 (12)	0.0028 (12)	−0.0166 (12)
C17	0.0400 (13)	0.0417 (13)	0.0701 (17)	−0.0152 (11)	−0.0069 (12)	−0.0076 (12)
N1	0.0288 (8)	0.0341 (9)	0.0271 (8)	−0.0091 (7)	−0.0001 (7)	−0.0014 (7)
O1	0.0290 (7)	0.0560 (10)	0.0274 (7)	−0.0179 (7)	−0.0024 (6)	−0.0014 (7)
O2	0.0281 (7)	0.0587 (10)	0.0355 (8)	−0.0200 (7)	0.0042 (6)	−0.0064 (7)
O3	0.0414 (9)	0.0763 (12)	0.0367 (9)	−0.0303 (9)	−0.0009 (7)	0.0050 (8)
O4	0.0618 (12)	0.0754 (13)	0.0467 (10)	−0.0448 (10)	0.0172 (9)	−0.0239 (9)
O5	0.0268 (8)	0.0655 (11)	0.0470 (9)	−0.0145 (8)	−0.0050 (7)	0.0094 (8)
O6	0.0350 (8)	0.0511 (10)	0.0497 (10)	−0.0179 (7)	0.0021 (7)	−0.0060 (8)
S1	0.0338 (3)	0.0366 (3)	0.0427 (3)	−0.0104 (2)	−0.0032 (2)	0.0048 (2)
Zn1	0.02999 (15)	0.05017 (18)	0.02839 (14)	−0.01813 (11)	−0.00285 (9)	0.00044 (10)

### Geometric parameters (Å, °)

**Table:**

C1—C6	1.386 (3)	C12—H12	0.9300
C1—C2	1.393 (3)	C13—C14	1.376 (4)
C1—C7	1.519 (3)	C13—H13	0.9300
C2—C3	1.388 (4)	C14—C15	1.418 (3)
C2—H2	0.9300	C14—H14	0.9300
C3—C4	1.383 (5)	C15—O3	1.310 (3)
C3—H3	0.9300	C16—S1	1.777 (3)
C4—C5	1.361 (5)	C16—H16A	0.9600
C4—H4	0.9300	C16—H16B	0.9600
C5—C6	1.393 (4)	C16—H16C	0.9600
C5—H5	0.9300	C17—S1	1.780 (3)
C6—H6	0.9300	C17—H17A	0.9600
C7—N1	1.469 (3)	C17—H17B	0.9600
C7—C8	1.544 (3)	C17—H17C	0.9600
C7—H7	0.9800	N1—Zn1	2.0305 (19)
C8—O1	1.248 (2)	O1—Zn1	2.1047 (17)
C8—O2	1.248 (2)	O3—Zn1	1.9742 (18)
C9—N1	1.276 (3)	O4—Zn1	2.009 (2)
C9—C10	1.443 (3)	O4—H23	0.8500
C9—H9	0.9300	O4—H24	0.8500
C10—C11	1.405 (3)	O5—Zn1	2.0015 (19)
C10—C15	1.430 (3)	O5—H21	0.8500
C11—C12	1.365 (4)	O5—H22	0.8499
C11—H11	0.9300	O6—S1	1.5147 (18)
C12—C13	1.382 (5)		
			
C6—C1—C2	119.6 (2)	C13—C14—C15	121.7 (3)
C6—C1—C7	119.8 (2)	C13—C14—H14	119.2
C2—C1—C7	120.64 (19)	C15—C14—H14	119.2
C3—C2—C1	119.6 (3)	O3—C15—C14	118.8 (2)
C3—C2—H2	120.2	O3—C15—C10	124.8 (2)
C1—C2—H2	120.2	C14—C15—C10	116.4 (2)
C4—C3—C2	120.3 (3)	S1—C16—H16A	109.5
C4—C3—H3	119.9	S1—C16—H16B	109.5
C2—C3—H3	119.9	H16A—C16—H16B	109.5
C5—C4—C3	120.2 (2)	S1—C16—H16C	109.5
C5—C4—H4	119.9	H16A—C16—H16C	109.5
C3—C4—H4	119.9	H16B—C16—H16C	109.5
C4—C5—C6	120.4 (3)	S1—C17—H17A	109.5
C4—C5—H5	119.8	S1—C17—H17B	109.5
C6—C5—H5	119.8	H17A—C17—H17B	109.5
C1—C6—C5	119.9 (3)	S1—C17—H17C	109.5
C1—C6—H6	120.1	H17A—C17—H17C	109.5
C5—C6—H6	120.1	H17B—C17—H17C	109.5
N1—C7—C1	111.97 (16)	C9—N1—C7	119.69 (18)
N1—C7—C8	108.26 (15)	C9—N1—Zn1	124.24 (15)
C1—C7—C8	109.72 (17)	C7—N1—Zn1	115.85 (13)
N1—C7—H7	108.9	C8—O1—Zn1	116.45 (13)
C1—C7—H7	108.9	C15—O3—Zn1	125.28 (15)
C8—C7—H7	108.9	Zn1—O4—H23	121.1
O1—C8—O2	124.70 (19)	Zn1—O4—H24	117.3
O1—C8—C7	118.68 (17)	H23—O4—H24	109.4
O2—C8—C7	116.62 (18)	Zn1—O5—H21	118.0
N1—C9—C10	126.4 (2)	Zn1—O5—H22	108.9
N1—C9—H9	116.8	H21—O5—H22	109.0
C10—C9—H9	116.8	O6—S1—C16	104.74 (13)
C11—C10—C15	119.5 (2)	O6—S1—C17	106.13 (13)
C11—C10—C9	116.5 (2)	C16—S1—C17	98.10 (13)
C15—C10—C9	124.1 (2)	O3—Zn1—O5	97.31 (8)
C12—C11—C10	122.4 (3)	O3—Zn1—O4	96.26 (9)
C12—C11—H11	118.8	O5—Zn1—O4	118.61 (9)
C10—C11—H11	118.8	O3—Zn1—N1	92.18 (8)
C11—C12—C13	118.6 (3)	O5—Zn1—N1	119.53 (8)
C11—C12—H12	120.7	O4—Zn1—N1	119.38 (9)
C13—C12—H12	120.7	O3—Zn1—O1	171.33 (6)
C14—C13—C12	121.5 (3)	O5—Zn1—O1	87.49 (8)
C14—C13—H13	119.3	O4—Zn1—O1	87.69 (7)
C12—C13—H13	119.3	N1—Zn1—O1	79.16 (7)
			
C6—C1—C2—C3	−1.5 (4)	C11—C10—C15—C14	0.0 (3)
C7—C1—C2—C3	178.5 (2)	C9—C10—C15—C14	−179.7 (2)
C1—C2—C3—C4	0.2 (4)	C10—C9—N1—C7	−178.51 (19)
C2—C3—C4—C5	0.9 (5)	C10—C9—N1—Zn1	−4.2 (3)
C3—C4—C5—C6	−0.8 (5)	C1—C7—N1—C9	−77.4 (2)
C2—C1—C6—C5	1.6 (4)	C8—C7—N1—C9	161.56 (19)
C7—C1—C6—C5	−178.4 (2)	C1—C7—N1—Zn1	107.87 (16)
C4—C5—C6—C1	−0.5 (4)	C8—C7—N1—Zn1	−13.2 (2)
C6—C1—C7—N1	126.6 (2)	O2—C8—O1—Zn1	174.12 (17)
C2—C1—C7—N1	−53.4 (3)	C7—C8—O1—Zn1	−7.0 (2)
C6—C1—C7—C8	−113.2 (2)	C14—C15—O3—Zn1	−165.10 (19)
C2—C1—C7—C8	66.9 (3)	C10—C15—O3—Zn1	16.3 (3)
N1—C7—C8—O1	13.2 (3)	C15—O3—Zn1—O5	−139.4 (2)
C1—C7—C8—O1	−109.3 (2)	C15—O3—Zn1—O4	100.7 (2)
N1—C7—C8—O2	−167.87 (18)	C15—O3—Zn1—N1	−19.2 (2)
C1—C7—C8—O2	69.7 (2)	C9—N1—Zn1—O3	13.31 (19)
N1—C9—C10—C11	174.9 (2)	C7—N1—Zn1—O3	−172.18 (14)
N1—C9—C10—C15	−5.4 (4)	C9—N1—Zn1—O5	112.98 (18)
C15—C10—C11—C12	1.0 (4)	C7—N1—Zn1—O5	−72.52 (15)
C9—C10—C11—C12	−179.3 (3)	C9—N1—Zn1—O4	−85.13 (19)
C10—C11—C12—C13	−0.9 (5)	C7—N1—Zn1—O4	89.38 (15)
C11—C12—C13—C14	−0.2 (5)	C9—N1—Zn1—O1	−166.21 (19)
C12—C13—C14—C15	1.2 (5)	C7—N1—Zn1—O1	8.29 (13)
C13—C14—C15—O3	−179.8 (3)	C8—O1—Zn1—O5	120.12 (16)
C13—C14—C15—C10	−1.1 (4)	C8—O1—Zn1—O4	−121.10 (16)
C11—C10—C15—O3	178.6 (2)	C8—O1—Zn1—N1	−0.59 (15)
C9—C10—C15—O3	−1.1 (4)		

### Hydrogen-bond geometry (Å, °)

**Table:**

*D*—H···*A*	*D*—H	H···*A*	*D*···*A*	*D*—H···*A*
O4—H23···O6	0.85	1.90	2.725 (3)	164
O4—H24···O2^i^	0.85	1.88	2.695 (3)	160
O5—H21···O2^ii^	0.85	1.81	2.629 (2)	161
O5—H22···O6^iii^	0.85	1.93	2.742 (3)	159

Symmetry codes: (i) −*x*+2, −*y*+1, −*z*; (ii) *x*−1, *y*, *z*; (iii) −*x*+1, −*y*+1, −*z*.
